# Primary Human Tissue Models for Metabolic Dysfunction‐Associated Liver Disease ‐ toward Streamlining Drug Discovery with Patient‐Derived Assays

**DOI:** 10.1002/adbi.202500337

**Published:** 2025-11-07

**Authors:** Sonia Youhanna, Nayere Taebnia, Yingxin Liang, Ningtao Cheng, Yi Wang, Maurice Michel, Volker M. Lauschke

**Affiliations:** ^1^ Department of Physiology and Pharmacology Karolinska Institutet Stockholm 17165 Sweden; ^2^ Center for Molecular Medicine Karolinska Institutet and University Hospital Stockholm 17176 Sweden; ^3^ HepaPredict AB Stockholm 17165 Sweden; ^4^ College of Pharmaceutical Sciences Zhejiang University Hangzhou 310058 China; ^5^ School of Public Health Zhejiang University School of Medicine Zhejiang 310058 China; ^6^ The Key Laboratory of Intelligent Preventive Medicine of Zhejiang Province Hangzhou 310058 China; ^7^ Science for Life Laboratory Department of Oncology‐Pathology Karolinska Institutet Stockholm 17164 Sweden; ^8^ Dr Margarete Fischer‐Bosch Institute of Clinical Pharmacology 70376 Stuttgart Germany; ^9^ University of Tübingen 72074 Tübingen Germany; ^10^ Department of Pharmacy the Second Xiangya Hospital Central South University Changsha 410013 China

**Keywords:** MASH, MASLD, microphysiological systems, organoids, patient‐derived, spheroids

## Abstract

Metabolic dysfunction‐associated steatotic liver disease (MASLD) and its progressive form metabolic dysfunction‐associated steatohepatitis (MASH) are prevalent chronic liver diseases that are closely linked to metabolic syndrome, type 2 diabetes, and cardiovascular complications. Despite their rising incidence and growing socioeconomic burden, effective therapies remain limited. Traditional preclinical models often fail to replicate the complexity of human MASLD, particularly in capturing the interplay between patient‐specific predisposition, metabolic dysfunction, immune activation and progressive fibrosis. In this review, a comprehensive overview of emerging human‐based in vitro and ex vivo platforms is provided for use in MASLD research, including conventional 2D cultures, organoids, 3D spheroids, precision‐cut liver slices, microphysiological systems, and bioprinted constructs. Their utility is evaluated for modeling different stages of MASLD and MASH and their alignment with key disease hallmarks is discussed. Furthermore, the different models are assessed for their capability to model pathophysiologically relevant nutritional exposure, to emulate genetic risk factors, to reflect the complex hepatic cell repertoire and to conduct high‐throughput drug screenings. Recent successful applications of MASLD and MASH models are highlighted in drug discovery and development. Together, these insights aim to guide the refinement of human MASLD models to narrow the translational gap in MASH drug development.

## Graphical abstract

The review provides a critical up‐to‐date overview of the current landscape of human in vitro models for fatty liver disease, including spheroids, organoids, organ‐on‐a‐chip systems, bioprinted liver constructs and precision‐cut liver slices. Their utility for faithfully modeling different stages of MASLD and MASH are evaluated and their compatibility with different applications is discussed.

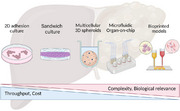

## 1 Introduction

Metabolic dysfunction‐associated steatotic liver disease (MASLD) is the newly adopted terminology for what was previously known as non‐alcoholic fatty liver disease (NAFLD). The diagnostic criteria of MASLD include hepatic steatosis plus at least one cardiometabolic risk factor, such as obesity, hypertriglyceridemia, hypertension, low levels of HDL cholesterol or high blood glucose in the absence of secondary causes of steatosis. Thereby, this new definition shifts the focus away from a simple description of hepatic fat accumulation to the recognition of metabolic dysfunction as the primary disease driver. MASLD represents a spectrum of diseases, ranging from simple and reversible steatosis to metabolic dysfunction‐associated steatohepatitis (MASH), fibrosis and end‐stage liver diseases including cirrhosis and hepatocellular carcinoma (HCC).^[^ ^1^, ^2^ ^]^ Disease progression is influenced by a combination of physiological, pathophysiological, environmental and lifestyle factors, including poor dietary habits, sedentary behavior, hypertension, sleep apnea, abdominal obesity and hyperlipidemia.^[^ ^3^, ^4^ ^]^ Additionally, polymorphisms in multiple genes such as *PNPLA3*, *TM6SF2*, and *MBOAT7* have been identified as genetic risk factors for MASLD onset and progression.^[^ ^5^, ^6^ ^]^ Furthermore, recent evidence suggests that polygenic architectures can stratify MASLD into different subtypes, such as a more aggressive disease confined to the liver and a more systemic disease associated with worse cardiometabolic outcomes.^[^ ^7^ ^]^

During the development and progression of MASLD both parenchymal and non‐parenchymal hepatic cell types undergo profound alterations in molecular phenotypes, metabolism and functionality.^[^ ^8^, ^9^ ^]^ One of the earliest and defining symptoms of MASLD is the accumulation of triglycerides within small intracellular lipid droplets within hepatocytes, a phenomenon called hepatic steatosis. While triglycerides themselves are neutral and non‐reactive, their buildup can reflect excess free fatty acids that cause lipotoxicity. These effects are morphologically paralleled by hepatocyte ballooning, which contributes to the activation of liver resident macrophages (Kupffer cells), the recruitment of infiltrating immune cells and the generation of an overall pro‐inflammatory milieu.^[^ ^10^ ^]^ This progression to MASH coincides with the progressive activation of hepatic stellate cells, resulting in their transformation from normally quiescent liver pericytes into fibrogenic αSMA^+^ myofibroblasts, the key players in the formation of hepatic fibrosis by virtue of depositing excessive extracellular matrix (ECM) components such as collagens.^[^ ^11^ ^]^ These pathogenic processes are modulated by liver sinusoidal endothelial cell (LSEC) dysfunction, manifesting in reduced fenestrations and altered signaling, which further aggravates inflammation and stellate cell activation.^[^ ^12^ ^]^ This complex interplay is paramount for the progression of simple steatosis to MASH and fibrosis.

MASLD is frequently associated with metabolic syndrome (MetS), a cluster of interrelated conditions including obesity, insulin resistance, hypertension and dyslipidemia. The connection between MASLD and MetS is well‐documented, with studies demonstrating that 41% of individuals diagnosed with MASLD also meet the clinical criteria for MetS.^[^ ^13^ ^]^ In addition to being a key factor in MASLD etiology, the presence of MetS also exacerbates disease progression. Insulin resistance in particular plays an important role in the pathogenesis and progression of MASLD. Studies indicate that 60% of patients with type 2 diabetes mellitus (T2DM) suffer from MASLD, while 15% of these patients develop advanced fibrosis.^[^ ^14^, ^15^ ^]^ These interactions highlight that systemic metabolic dysfunction is central to the pathogenesis and consequences of MASLD. Mechanistically, insulin resistance contributes to disease progression by promoting hepatic lipogenesis, inflammation, and oxidative stress.^[^ ^16^, ^17^ ^]^ Furthermore, the presence of T2DM significantly increases the risk of HCC, the most severe hepatic complication of MASLD.^[^ ^18^ ^]^

MASLD has become the globally most prevalent liver disease affecting an estimated 38% of the adult population worldwide in 2020.^[^ ^19^ ^]^ Furthermore, recent forecasts suggest that MASLD prevalence will further increase to 41‐55% of the adult population over the next two decades.^[^ ^19^, ^20^ ^]^ The increasing prevalence of MASLD is a significant concern for healthcare systems worldwide, as it leads to a growing number of patients requiring long‐term management and, in case of end‐stage liver disease, liver transplantation. These measures result in high health care costs with annual direct medical costs exceeding 100 billion in the US and 35 billion EUR in Europe.^[^ ^21^ ^]^ Notably, hypothetical pharmacological treatments that could halt fibrosis progression promise to reduce direct healthcare costs by 40.5–99 billion USD, whereas therapy that only reverses steatosis was predicted to have only minimal socioeconomic effects.^[^ ^22^ ^]^

Despite its rising prevalence, treatment options for MASLD remain limited. Currently, only two pharmacological treatments have been FDA‐approved. Resmetirom, a thyroid hormone receptor β‐selective agonist is effective in reducing hepatic steatosis and fibrosis; however, only 25% of patients showed an improvement of fibrosis after 52 weeks of treatment in the pivotal MAESTRO‐NASH phase 3 randomized controlled trial.^[^ ^23^ ^]^ Recently, semaglutide received approval for MASH and showed improvement in fibrosis in 37% of patients.^[^ ^24^ ^]^ A key challenge in MASLD treatment development is the heterogeneity of the disease, which requires personalized treatment approaches that target both hepatic manifestations as well as extrahepatic metabolic dysfunction and there remains an urgent need for the development of further pharmacotherapies.^[^ ^25^, ^26^ ^]^

The limited availability of therapeutic options highlights critical gaps in MASLD drug discovery and development. Conventional preclinical models have contributed valuable insights into MASLD pathogenesis but fail to fully recapitulate the complexity of human disease. Yet, rodent models commonly do not capture the spectrum of human MASLD and lack the diversity and inter‐individual variability of the human patient population.^[^ ^27^ ^]^ On the other hand, in vitro systems based on cell lines or primary human hepatocyte monocultures do not reflect the molecular phenotypes of liver cells in situ and fail to represent the complexity of cellular interactions that are an integral feature of MASH and fibrosis. This discrepancy highlights the need for better translational models that accurately reflect MASLD etiology and progression.

Emerging human‐relevant models, such as patient‐derived liver spheroids, organoids, explant cultures and microfluidic systems, offer promising opportunities for improving disease modeling and accelerating drug discovery. These platforms aspire to provide a more physiologically relevant environment to study MASLD pathobiology, identify novel biomarkers and therapeutic targets and assess drug efficacy. Additionally, integrating multi‐omics approaches (genomics, transcriptomics, proteomics, and metabolomics) alongside bioinformatics and artificial intelligence into disease modeling can enhance our understanding of MASLD heterogeneity and enable the development of more precise and personalized therapies.

## 2 What Makes a Good In Vitro MASLD Model?

Developing accessible in vitro model systems that accurately replicate the critical hallmarks of human MASLD is essential for translational research into the disease. An ideal disease model should meet several key requirements:

1. Cultured cells must closely mimic the molecular and cellular phenotypes of their respective in vivo counterparts. In addition, the cellular composition should reflect the composition of the liver, including not only hepatocytes but also non‐parenchymal cells (NPCs). Phenotypes must be stable for extended periods of time (i.e., at least multiple weeks).
2. The culture medium should be well‐defined, fully disclosed and allow for nutritional manipulation by changing carbohydrate, hormone and fatty acid levels. Ideally, cultures should be serum‐free and not rely on the extrinsic addition of inflammatory mediators (cytokines and chemokines) or stimuli (lipopolysaccharides) to emulate a pro‐inflammatory milieu.
3. The model should be extensively benchmarked against liver tissue in situ at the molecular, metabolic and functional level to characterize which MASLD features are accurately reflected (and which are not). Furthermore, assays should be standardized and reproducible.
4. Culture platforms should be fabricated from lowly absorbing materials to avoid interference with pharmacological and toxicological applications.
5. The culture format should be compatible with high‐throughput screening (HTS) to facilitate drug discovery efforts.

Jointly, these criteria ensure that experimental outcomes are reliable, translatable, and applicable to human health. In the following, we elaborate further on each of these requirements.

### 2.1 Molecular and Cellular Phenotypes and Complexity

**Figure 1:**
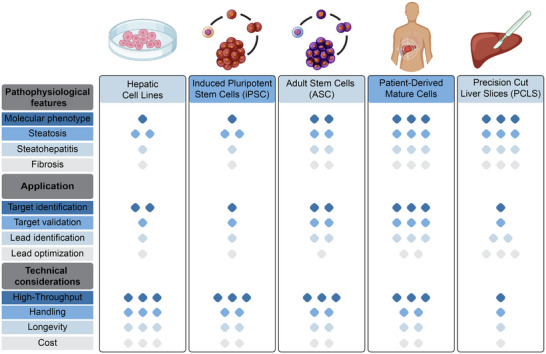
Comparative assessment of key cellular sources to model MASLD and MASH in vitro. The schematic highlights the strengths and limitations of commonly used cell models used in MASLD and MASH research, including hepatic cell lines, hepatocyte‐like cells derived from induced pluripotent stem cells (iPSCs) or adult stem cells (ASCs), patient‐derived mature cells and precision‐cut liver slices (PCLS). Higher number of diamonds indicates a more beneficial effect for the given parameter.

A physiologically relevant model should accurately reflect the cellular phenotype at the morphological, molecular and functional level, as well as capture the complexity of cellular interactions with other liver resident cells and the extracellular microenvironment. Cell models range from immortalized cell lines to stem cell‐derived hepatocyte‐like cells (HLCs), primary mature human liver cells and precision cut liver slices that each have specific advantages and limitations (**Figure** **1**). In vitro models based on immortalized cell lines are inexpensive and simple to use; however, they do not closely resemble primary human hepatocytes (PHH) at the transcriptomic, proteomic or metabolomic level.^[^ ^28^, ^29^, ^30^, ^31^, ^32^ ^]^ As alternatives, hepatocyte differentiation protocols based on induced pluripotent stem cells (iPSCs) or adult stem cells (ASCs) have been developed.^[^ ^33^, ^34^ ^]^ These cells can be propagated, genetically manipulated using genomic editors, which allows to study the functionality of candidate variants implicated in MASLD, such as *PNPLA3* p.I148M or variants in *APOB* or *MTTP*.^[^ ^35^, ^36^ ^]^ Furthermore, iPSCs can be generated from easily accessible cell populations directly from specific patients without the need of invasive procedures, thereby allowing, in principle, to recapitulate the effects of complex polygenic risk factors. However, the establishment of stable stem cell lines can be problematic^[^ ^37^ ^]^ and, while these protocols have seen improvements over the past decade with the resulting HLCs expressing key hepatic markers, such as albumin and cytochrome P450s, terminal differentiation has not yet been achieved, and the phenotypes of HLCs remain fetal.^[^ ^38^, ^39^, ^40^ ^]^

Organoid culture that generates self‐organizing 3D structures derived from obligate or facultative stem cells through cell‐cell and cell‐matrix interactions, constitutes a major advancement for hepatic stem cell differentiation. Whereas the aforementioned protocols used a series of different cocktails to drive HLC differentiation in conventional 2D culture, organoid culture combines the exposure to extrinsic biochemical cues with 3D culture in different hydrogels. A plethora of different protocols have been described; for methodological details, we refer the interested reader to recent comprehensive reviews on this topic.^[^ ^41^, ^42^ ^]^ In the context of MASLD, there are two distinct experimental strategies for the use of organoids. First, organoids can be exposed to different nutritional cues to induce MASLD in vitro or organoids can be formed directly from liver cells isolated from patients with an established MASLD diagnosis. However, it is important to highlight that most hepatic organoid models only generate hepatocyte‐like cells from iPSCs but not representatives of NPCs.^[^ ^43^, ^44^, ^45^, ^46^ ^]^ This limits their utility to the assessment of steatosis and hepatocellular stress but excludes the modeling of disease progression or the evaluation of inflammatory and fibrotic mechanisms. Furthermore, the molecular phenotypes even of complex vascularized organoids remains clearly distinct from primary adult human liver cells.^[^ ^47^ ^]^

Primary human liver cells (PHH and NPCs) isolated from organ donations or liver resections remain the gold‐standard model for the in vitro emulation of liver function. The different cell populations can be isolated via collagenase perfusion and subsequent size fractionation^[^ ^48^ ^]^ and the different fractions can be aliquoted and cryopreserved,^[^ ^49^ ^]^ allowing for the generation of patient‐derived liver cell biobanks. Upon thawing, cells are seeded in ultra‐low attachment plates where they aggregate to form uniform 3D clusters. These structures, commonly referred to as “liver spheroids”,^[^ ^50^ ^]^ are conceptually different from organoids in that they are comprised of fully differentiated mature cells that can be cultured in basal media, whereas organoids are derived from stem cells that are expanded and differentiated – often only partially – via complex exposure regimens involving mitogens and other media supplements. Under normal culture conditions, these mature liver cells do not divide, and the cellular material is thus finite; however, given that the human liver contains ≈100 billion cells, whereas typical 3D liver cultures contain only 500–5000 cells, this allows for a huge number of replicates for each donor. Furthermore, different strategies have recently been presented that can trigger proliferation of differentiated PHH opening further possibilities for cell expansion.^[^ ^51^, ^52^, ^53^ ^]^

While conventional 2D monolayer cultures rapidly lose liver‐specific functions within hours, 3D culture of primary human liver cells allows for the accurate maintenance of the molecular and metabolic phenotypes of the cultured cells for at least five weeks as evidenced by longitudinal transcriptomic, proteomic, metabolomic and functional data.^[^ ^54^, ^55^, ^56^ ^]^ Furthermore, unpublished data from our group indicate that hepatocytes can even be maintained for up to 16 weeks in spheroid culture. Exposure of liver spheroids comprised of PHH and NPCs to free fatty acids and high glucose levels allows for the investigation of disease‐specific cellular responses, such as lipid metabolism, oxidative stress, inflammatory signaling and fibrosis.^[^ ^57^, ^58^, ^59^, ^60^, ^61^, ^62^ ^]^ While efficient genetic editing in these models is at present not possible due to the inability to expand positive recombinants, oligonucleotide‐based knockdowns offer experimental alternatives and have been successfully used to identify the functional importance of *PSD3* in human MASH.^[^ ^63^ ^]^ Furthermore, by using primary liver cells from patients with a histologically established diagnosis of MASH, spheroid cultures accurately emulate steatosis, inflammation and fibrosis and can be used to study the heterogeneity that exists between MASLD patients in an accessible in vitro setting.^[^ ^64^ ^]^ Particularly for chronic diseases, such as MASLD, the use of patient‐derived cells is important as cellular phenotypes are shaped by years of nutritional insults. When mature patient‐derived cells are used, this history is naturally reflected, whereas conventional protocols that rely on in vitro high‐nutrient exposure of liver cells isolated from non‐diseased individuals for a few days to weeks cannot accurately recapitulate these chronic effects.

### 2.2 Nutritional Control

Nutritional factors play a key role in disease modeling and drug responses, particularly in metabolic diseases such as in MASLD. As such, it is important from an experimental perspective to be able to control and dynamically adjust nutrient and hormone levels in the culture milieu. Most culture methods for mature liver cells use basal serum‐free media compositions on a Williams E basis supplemented only with glutamine, transferrin, selenium and low levels of corticosteroids. If serum is used, it is critical that sources and concentrations are fully disclosed. By using a glucose‐free medium base, this allows for the exact and tailored manipulation of nutritional conditions. Steatosis is most commonly induced by a combination of high carbohydrate levels (glucose with or without fructose), high levels of insulin and albumin‐conjugated free fatty acids (FFAs), typically oleic and palmitic acid. Effects at the molecular, cellular and functional level are then compared to controls, which are cultured in a milieu with quasi‐physiological levels of insulin and glucose without exposure to FFAs. Quantification of glucose uptake kinetics upon insulin stimulation in such chemically defined culture conditions revealed for instance that glucose uptake in 3D human spheroids closely resembled human hepatic glucose uptake in vivo as measured by euglycemic‐hyperinsulinemic clamps.^[^ ^65^ ^]^ Notably, insulin concentrations are the main changing hormonal parameter in controls whereas glucagon or incretins are rarely included. In addition to nutrients and hormones, some models also include inflammatory factors, such as recombinant IL6, TGFβ or TNFα. However, an intrinsic regulation of inflammatory signaling via co‐cultured immune cells is typically preferred. Importantly, the use of commercial proprietary media significantly impairs this metabolic control and hampers mechanistic interpretations as the individual media components are not disclosed; moreover, commercial non‐disclosed media often perform worse in direct side‐by‐side comparisons.^[^ ^66^, ^67^ ^]^

Besides nutrients and hormones, exposure to gut‐derived factors constitutes an emerging area of research. For instance, application of gut microbiota‐derived metabolites, such as short‐chain fatty acids, to human liver cultures allows to explore their impact on hepatic metabolic phenotypes.^[^ ^68^ ^]^ The use of LPS is more controversial. In MASLD, gut‐derived LPS increasingly travel through the portal vein to the liver where they can trigger or exacerbate inflammation.^[^ ^69^ ^]^ Thus, co‐stimulation of MASLD cultures could be seen as a disease‐adequate pro‐inflammatory stimulus. Nevertheless, the use of LPS in MASLD cultures in vitro is limited despite its clear effects due to challenges associated with dosing. In the past, studies used highly supraphysiological levels, which then overshadowed more subtle increases in inflammation due to lipotoxicity. However, we believe that supplementation of MASLD cultures with careful low‐dose regimens LPS might be worth to revisit.

### 2.3 Model Characterization and Benchmarking

A well‐characterized model ensures reliable and reproducible results, requiring thorough validation of histological endpoints, biochemical markers and functional parameters. In the case of MASH samples, important parameters include the evaluation of basic hepatic features (albumin and urea production, expression of CYP3A4, CYP2D6, UGT1A1, ABCB11 and HNF4A), cellular composition of spheroids including cellular subtypes by immunostaining or FACS, insulin sensitivity by Western blot for phosphorylated AKT, apolipoprotein secretion, glucose uptake, steatosis as measured by imaging or quantification using the Adipored assay, inflammatory markers (e.g., IL6, IL8, TNFα) quantified by ELISA and fibrosis by staining for collagen 1A1. Furthermore, comprehensive omics profiling using transcriptomic, proteomics and metabolomic techniques provides important insights into the overall differentiation state and molecular configuration of the cultured cells. Lastly, careful profiling of the responses to pharmacological reference drugs is necessary to increase confidence in the translatability of new findings. All measured parameters should be benchmarked against the relevant reference material, for instance by using isogenic freshly isolated hepatocytes from the same donor as comparator. Furthermore, evaluation in time‐series experiments is important to confirm model stability over longer periods of time.

In addition to in‐depth comparisons against reference material, models and assays should be carefully characterized with regards to experimental reproducibility. For models based on cell lines or stem cells, stability over different passages and the possibly resulting drift should be assessed. For primary liver cells, cryopreservation allows for (a finite number of) replicate experiments using cellular material from the same donor. Furthermore, inter‐individual variability should be assessed for key functional endpoints. Specifically, for patient‐derived models it is critical to characterize the system to the point that inter‐individual differences can be distinguished for inter‐experimental variability. Only then can such systems be fit for personalized applications. Ideally, these benchmarking experiments should be conducted in multi‐center experiments using standardized protocols to facilitate conclusions regarding the variability between replicates and laboratories. While laborious, such extensive validation efforts are imperative before a novel platform can be more widely adopted for preclinical applications.

### 2.4 Drug Absorption of Culture Materials

An often‐overlooked factor in the design of organotypic and microphysiological models for preclinical applications is the absorption of small molecules into the material of the culture platforms. This issue is particularly relevant for MPS and organ‐on‐chip platforms, where commonly used polymers such as polydimethylsiloxane (PDMS) have long been known to significantly reduce the free concentration of test compounds.^[^ ^70^ ^]^ While PDMS remains the most commonly used material for microfluidic devices, an increasing number of systems are fabricated from other biocompatible thermoplastic and thermosetting materials, such as poly(methyl methacrylate), polycarbonate, cyclic olefin copolymer, and thiol‐ene epoxy.^[^ ^71^ ^]^ Comparison of drug absorption in polymer devices with identical designs fabricated from different materials revealed that absorption differed by over 1000‐fold.^[^ ^72^ ^]^ PDMS was shown to be the most absorptive material, whereas polytetrafluoroethylene (Teflon) and thiol‐ene epoxy exhibited the lowest absorption. Absorption was predominantly determined by compound hydrophobicity (logP > 2.5), the number of hydrogen donors and rotatable bonds, whereas the hydrophobicity of the polymer itself did not predict absorption.^[^ ^72^, ^73^, ^74^ ^]^ When used for toxicological assessments, absorption drastically impacted preclinical risk assessments, resulting in false negatives for test articles that were hydrophobic, such as chlorpromazine and tamoxifen.^[^ ^72^ ^]^ These findings underscore the importance of material selection to increase the translational accuracy of hepatic in vitro assays.

### 2.5 Compatibility with High‐Throughput Applications

For drug discovery, it is helpful if the established models are compatible with conventional screening formats. This includes that the models can be cultured in 96‐ or 384‐well plates and can be interfaced with robotic handling and imaging techniques. In iPSC‐derived HLC 2D cultures, a screen of 1120 biologically active compound resulted in the identification of the Src/PI3K/Akt signaling node as a potential anti‐steatotic target in PNPLA3 I148M carriers.^[^ ^75^ ^]^ In 3D liver spheroids, multiplexed chemogenomic screens of >100 compounds revealed the CHRM1‐TRPM8 axis as a new target for hepatic fibrosis^[^ ^64^ ^]^ and showed that epigenetic plasticity was an essential prerequisite for hepatocyte regeneration.^[^ ^76^ ^]^ In liver organoids, screening platforms have been mostly developed to test the hepatotoxicity of drugs and drug candidates where automatic systems can evaluate biochemical assays for albumin and serum transferases, and microscopy‐based morphological profiling in parallel.^[^ ^77^, ^78^ ^]^ Due to their self‐organizing properties, the variability between organoids is substantially larger than variability between spheroids, resulting in lower Z′‐factors. Screening costs are another important factor that should be taken into consideration when planning high‐throughput applications since the utilized cells and reagents can present significant cost‐drivers. In this context, microfluidic and bioprinting technologies can help to lower cost by miniaturizing assays and reducing reagent volumes.^[^ ^79^ ^]^ However, more work is required to interface these technologies with the screening in complex 3D MASH organoid or spheroid models.

## 3 Overview of the Available Human In Vitro Models for MASLD and MASH

In recent years, the field of liver disease research has witnessed a substantial shift away from 2D cell cultures and toward the development of organotypic, more physiologically relevant human in vitro models that better mimic the complex pathophysiology of MASLD. Two principally different sets of liver culture methods for MASLD can be distinguished: i) Assays that only include hepatocyte cell models but lack models of inflammatory cells or stellate cells. These models can only be used to study steatosis characteristic of early MASLD stages. ii) Systems that contain the full complement of cells and aspire to also model disease progression to MASH and fibrosis.

### 3.1 Model Systems for Hepatic Steatosis and Early MASLD

Different models for early MASLD have been presented (**Table** **1**). For these systems, cells are typically cultured in 2D monolayers or organoids that lack non‐parenchymal cells. Immortalized hepatic cell lines, such as HepG2, Huh7 and HepaRG have a long history for in vitro investigations of liver metabolism. HepG2 and Huh7 are derived lines from hepatocellular carcinomas whereas HepaRG cells originate from a liver tumor associated with chronic hepatitis C and haemochromatosis. Notably, careful selection of cell lines is of critical importance for the generation of reliable results due to potential risks of contamination and misclassification of cell lines. For instance, HuL‐1 and WRL‐68 cells, which were believed to be of hepatic origin, were later identified to be cervical adenocarcinoma lines, likely from a HeLa cell‐derivative.^[^ ^80^ ^]^

**Table 1:** Overview of human in vitro models limited to early MASLD.

						MASLD endpoints					
Hepatocyte model	NPCs	Culture system	Matrix	Disease induction	Benchmarking to liver	Steatosis	Inflammation	Fibrosis	Omics	Drug response	Refs.
HepG2, PHH	None	Monolayer	None	PA, tunicamycin	None	✓					[86]
PHH, HepG2	None	Monolayer	None	FFA	None	✓					[81]
PHH, Huh7	None	Monolayer and 3D spheroid	None	OA	None	✓	✓				[63]
PHH	3T3‐J2 murine fibroblasts as supporting cells	MPCC	Collagen I	Hypo‐/Hyper‐glycemic media	None	✓				✓	[97]
PHH	None	Sandwich culture	Collagen	FFA	None	✓	✓		✓	✓	[100]
hiPSC‐derived HLC	None	Organoid	Matrigel	FFA	Benchmarking against MASLD liver by RNA‐Seq	✓	✓		✓	✓	[44]
ASC‐derived HLC	None	Organoid	Matrigel	None	Benchmarking against MASLD liver by RNA‐Seq	✓			✓		[45]
hiPSC‐derived HLC	None	Organoid	Matrigel	FFA	None	✓			✓		[35]
Human fetal hepatocyte organoids	None	Organoid	Matrigel	FFA, CRISPR	Benchmarking against MASLD liver by RNA‐Seq	✓			✓	✓	[36]
hiPSC‐derived HLC	None	Microfluidic chip	None	FFA	None	✓	✓				[120]

FFA = free fatty acids; NPC = non‐parenchymal cells; PA = palmitic acid; PHH = primary human hepatocyte; hiPSC = human induced pluripotent stem cell; ASC = adult stem cell; HLC = hepatocyte‐like cell; MPCC = micropatterned co‐culture.

In the context of MASLD, exposure to fatty acids can readily induce steatosis.^[^ ^81^, ^82^, ^83^ ^]^ Furthermore, the use of saturated fatty acids like palmitic acid can enhance oxidative stress and promote apoptosis, thereby simulating a transition toward lipotoxicity in progressing disease.^[^ ^84^, ^85^ ^]^ Cell lines have been used with some success to identify candidate factors involved in hepatocellular metabolism; for instance, exposure of HepG2 cells with the adiponectin paralog CTRP9, resulted in reduced steatosis and ameliorated palmitate‐induced endoplasmic reticulum stress – effects, which were overall confirmed in PHH.^[^ ^86^ ^]^

However, the highly dedifferentiated nature and altered karyotype of hepatoma cell lines limit their physiological relevance. Specifically, they lack the full complement of cytochrome P450 enzymes, exhibit fetal‐like transcriptional profiles, and have reduced responsiveness to key metabolic hormones such as insulin and glucagon. The HepaRG cell line partially overcomes these limitations. Upon differentiation, HepaRG cells exhibit improved hepatocellular marker expression and metabolic competence.^[^ ^87^, ^88^ ^]^ However, despite these advances, bioenergetics in proliferating cell lines remain fundamentally different compared to quiescent mature human hepatocytes, which limits the utility of hepatoma cells for studies of lipid metabolism.^[^ ^89^ ^]^

Directed differentiation of iPSCs or ASCs can yield HLCs that express hepatocyte markers, albeit generally at lower levels than primary hepatocytes, and accumulate features of hepatic steatosis, including intracellular lipid droplet accumulation upon FFA exposure and ER stress.^[^ ^44^, ^90^ ^]^ Furthermore, iPSC lines derived from MASH patients showed increased lipid accumulation and unique transcriptomic fingerprints linked to insulin resistance and oxidative stress, suggesting that they might be potentially useful for the investigation of polygenic predisposing factors. Genomic editing allows to introduce defined variants of interest into otherwise isogenic stem cell lines. For instance, genetic manipulation of *PNPLA3* in iPSCs – either by introducing the p.I148M variant or by knockout of the gene – resulted in increased steatosis in HLCs upon differentiation, suggesting that PNPLA3^I148M^ results in reduced function.^[^ ^35^ ^]^ Similar results were observed in a recent study using a perfused liver system based on primary hepatocytes in which the authors demonstrated that the p.I148M variant was associated with increased lipid accumulation.^[^ ^91^ ^]^ However, more recent findings in primary hepatocytes and mice suggest that the pro‐steatotic effect of the p.I148M variant is due to a gain‐of‐function effect; specifically, PNPLA3^I148M^ appears to accumulate on lipid droplets and inhibits ATGL‐mediated lipolysis of triglycerides, which, in turn, results in decreased hepatic triglyceride secretion.^[^ ^92^, ^93^ ^]^ These preclinical results are in line with the findings from ongoing clinical trials that indicate that hepatic downregulation of PNPLA3 rather than its activation ameliorates hepatic steatosis.^[^ ^94^ ^]^

PHHs remain the gold‐standard for studies of human liver cell biology. Upon stimulation with free fatty acids or elevated levels of glucose, they become steatotic, and exhibit elevated expression of lipogenic genes, IL8 secretion and signs of ER stress.^[^ ^95^, ^96^, ^97^, ^98^ ^]^ While PHH lose hepatic phenotypes in conventional 2D monolayer culture within hours,^[^ ^99^ ^]^ when cultured in appropriate 3D confirmation, they retain mature metabolic profiles and disease‐relevant transcriptomic signatures for multiple weeks.^[^ ^54^, ^55^ ^]^ Despite these limitations, 2D sandwich cultures remain in use for investigations of steatosis, insulin resistance and inflammation in MASLD.^[^ ^100^ ^]^ PHH can recapitulate the effects of important genetic risk factors of fatty liver, such as the effect of *TM6SF2* p.E167K on lipogenesis and apolipoprotein secretion.^[^ ^101^ ^]^ However, these approaches require the possibility to obtain primary liver cells from the respective variant carriers, which is often difficult or impractical for rare or ultra‐rare variants. Importantly, hepatocyte monocultures, be it from cell lines, stem cell‐derived HLCs or PHH, can only mimic the early stages of MASLD as they are limited to hepatocellular effects. To emulate disease progression more complex co‐culture models are required in which hepatocyte models are complemented with immune cells and stellate cells.

### 3.2 Model Systems for MASH and Fibrosis

A dysfunctional interplay between parenchymal and non‐parenchymal cell types, including immune cells,^[^ ^102^, ^103^ ^]^ hepatic stellate cells (HSCs)^[^ ^11^ ^]^ and LSECs^[^ ^104^ ^]^ underlies the metabolic dysregulation, inflammation and fibrosis observed in MASH. To capture these cell–cell interactions in vitro, co‐cultures are required in which hepatocyte models are complemented with immune cells and stellate cells (**Table** **2**). For instance, 2D co‐cultures of hepatocytes with the human stellate cell line LX2 have shown synergistic upregulation of TGFβ and αSMA upon FFA exposure, mimicking events during early fibrosis.^[^ ^105^, ^106^ ^]^ Similarly, micropatterned co‐cultures of PHH and activated primary human stellate cells resulted in increased steatosis and inflammatory signaling, which could be alleviated via clinically used FXR agonists.^[^ ^107^ ^]^ However, as described above, 2D cultures fail to maintain physiologically relevant phenotypes; consequently, the majority of MASH co‐culture models are based on 3D microtissues, such as organoids or spheroids.

**Table 2:** Overview of human in vitro and ex vivo models of MASH and fibrosis.

						MASLD endpoints					
Hepatocyte model	NPCs	Culture system	Matrix	Disease induction	Benchmarking to liver	Steatosis	Inflammation	Fibrosis	Omics	Drug response	Refs.
PHH, HepG2, Huh7	HSC	Monolayer	None	PA	None		✓	✓			[96]
L02	HUVEC	Monolayer	None	FFA	None		✓	✓			[151]
hSKP‐HLC, HepG2, HepaRG, PHH	LX‐2	Monolayer	Collagen I	FFA, TNFα, IL1β, TGFβ	None	✓	✓	✓		✓	[105]
PHH	LSEC	Monolayer	None	OA, SA	None	✓		✓			[152]
PHH	HSC	Monolayer	None	TGFβ, PA, H_2_O_2_	None		✓	✓			[153]
PHH	HSC	Cell sheets	Collagen and iMatrix	FFA	None	✓		✓			[154]
HepG2	LX‐2	3D spheroid	None	FFA	None	✓		✓			[155]
PHH	NPC	3D spheroid	None	FFA, TGFβ	None		✓	✓			[60]
C3A	LX‐2	3D spheroid	None	OA	None	✓	✓	✓			[156]
PHH	HSC, KC, LSEC	3D spheroid	None	LDL, LPS	None	✓	✓	✓	✓		[157]
HepG2	HUVEC	3D spheroid, microfluidic chip	Collagen	FFA	None	✓	✓			✓	[118]
PHH	NPC	3D spheroid	None	FFA	None	✓		✓		✓	[58]
HepG2, HepaRG	HUVEC, Kup5, KC	3D spheroid, microfluidic chip	Gelatin methacryloyl	FFA	None	✓	✓				[158]
PHH	HSC, LSEC, KC	Bioprinted constructs	NovoGel 2.0	None	Benchmarking against freshly isolated cells by single cell RNA‐Seq			✓			[137]
PHH	HSC and 3T3‐J2 murine fibroblasts as supporting cells	MPCC	Collagen I	Activated HSCs	None	✓	✓	✓		✓	[107]
PHH	HSC, KC	Microfluidic chip	Collagen I	Fructose, LPS, cholesterol, TGFβ	Benchmarking against control and MASH biopsies by RNA‐Seq	✓	✓	✓			[106]
hiPSC‐derived HLC	Human mesenchymal cells, fibroblasts, MΦ	Microfluidic chip	Decellularized rat liver ECM	FFA	Benchmarking against control and MASH biopsies by PCR array	✓	✓	✓	✓		[43]
hiPSC‐derived HLC, ESC derived HLC	Stellate‐like cells, Kupffer‐like cells	Organoid	Matrigel	FFA	Benchmarking against primary hepatocyte cells and fetal liver by scRNA‐Seq	✓	✓	✓	✓	✓	[108]
hiPSC‐derived HLC	Stellate‐like cells, Kupffer‐like cells	Organoid	Matrigel	FFA, high glucose, insulin	Benchmarking against primary hepatocyte cells by RNA‐Seq	✓	✓		✓	✓	[109]
hiPSC‐derived HLC	Stellate‐like cells	Organoid	Matrigel	FFA, TGFβ	Benchmarking against MASH liver public data by RNA‐seq	✓	✓	✓	✓	✓	[110]
HepaRG	LX‐2, LSEC	Microfluidic chip	Fibrin	FFA	None	✓				✓	[133]
PHH	KC, LSEC, HSC	Microfluidic chip	Collagen	FFA, LPS	None	✓	✓	✓		✓	[123]
PHH	HSC, KC, LSEC	3D spheroid	None	FFA	None	✓	✓	✓		✓	[61]
HepaRG	MΦ, LX‐2	3D spheroid	None	SA, OA	None	✓	✓	✓		✓	[159]
PHH	LSEC, KC, HSC	Bioprinted constructs	Thermo‐responsive hydrogel	Fructose, FFA, LPS	Benchmarking against control and MASH biopsies by RNA‐Seq	✓	✓		✓	✓	[136]
ASC‐derived HLC	HSC	Organoid	Matrigel	FFA	None	✓	✓	✓	✓	✓	[46]
PHH	KC, LSEC, HSC	3D spheroid	None	Fructose, FFA, LPS	None	✓	✓	✓	✓	✓	[59]
PHH	Patient‐derived NPC	3D spheroid	None	FFA	Benchmarking against control and MASH biopsies by RNA‐Seq, proteomics and lipidomics	✓	✓	✓	✓	✓	[64]

FFA = free fatty acids; KC = Kupffer cells; LSEC = Liver sinusoidal endothelial cell; HSC = hepatic stellate cell; LPS = lipopolysaccharide; MPS = microphysiological system; NPC = non‐parenchymal cells; OA = oleic acid; PA = palmitic acid; SA = stearic acid; LDL = low‐density lipoprotein; PHH = primary human hepatocyte; MΦ = macrophage; hSKP: human skin stem cell; hiPSC = human induced pluripotent stem cell; ASC = adult stem cell; HLC = hepatocyte‐like cell; ECM = extracellular matrix; MPCC = micropatterned co‐culture.

#### 3.2.1 Organoid and Spheroid Models

PSC‐derived liver organoids are generated through stepwise differentiation protocols. While most protocols generate only HLCs, more recent approaches can yield multiple hepatic lineages that also include cholangiocyte‐like, Kupffer cell‐like, and HSC‐like cells within a single 3D construct.^[^ ^108^, ^109^, ^110^ ^]^ Oleic acid‐treated PSC‐derived liver organoids exhibited significant lipid accumulation, pro‐inflammatory cytokine expression (e.g., TNFα, IL6 and IL8) and induction of fibrotic markers (e.g., αSMA and P3NP).^[^ ^108^ ^]^ Importantly, fibrogenesis was not only observed at the molecular level but could also be measured biomechanically through atomic force microscopy, which revealed significant correlations between organoid stiffness and disease severity. This integration of mechanical and molecular phenotyping marks an important step toward the definition of preclinical parameters that can serve as important proxies for the relevant clinical endpoints in potential later trials. Despite their promise, the utility of such self‐organizing organoids is hampered by their overall immature phenotypes and considerable heterogeneity between organoids, especially when organoids contain varying proportions of cell types or exhibit inconsistent maturation. Moreover, further metabolic and functional profiling is needed to confirm whether lipid handling, insulin signaling, and mitochondrial behavior accurately reflect in vivo MASLD progression.

Advancements in organoid engineering have enabled better mimicking of structural features of the native liver, resulting in hepatic organoids composed of hepatocytes and cholangiocytes that self‐organize into a bile canalicular core connected to cyst‐like cholangiocyte domains.^[^ ^44^ ^]^ This architecture allows for the study of tissue remodeling and ductular reactions during disease progression. Notably, treatment with free fatty acids induced a decline in the canaliculi network and a proportional increase in CK7^+^ and Ki67^+^ cholangiocytes, closely paralleling ductular proliferation observed in human MASH livers.^[^ ^111^ ^]^ However, the absence of non‐parenchymal cells, such as lymphocytes and stellate cells, limits the application of these organoids in modeling the immunometabolic liver injury and fibrosis.

To overcome some of the limitations associated with PSC‐derived organoids, researchers have turned to co‐culture spheroids assembled from functionally mature PHH and NPCs. Spheroids from non‐MASH individuals that integrate primary human hepatocytes, Kupffer cells, LSECs, and HSCs in scaffold‐free suspension cultures mimicked steatosis, inflammation and fibrotic marker expression upon exposure to media supplemented with free fatty acids, glucose and fructose.^[^ ^58^, ^61^, ^112^ ^]^ Such models are well‐suited to study the etiology of MASH and have been demonstrated to recapitulate responses to therapeutic candidate molecules, such as firsocostat, selonsertib, elafibranor and lanifibranor. However, they rely on the induction of MASH phenotypes in vitro, which might limit translational relevance.

**Figure 2:**
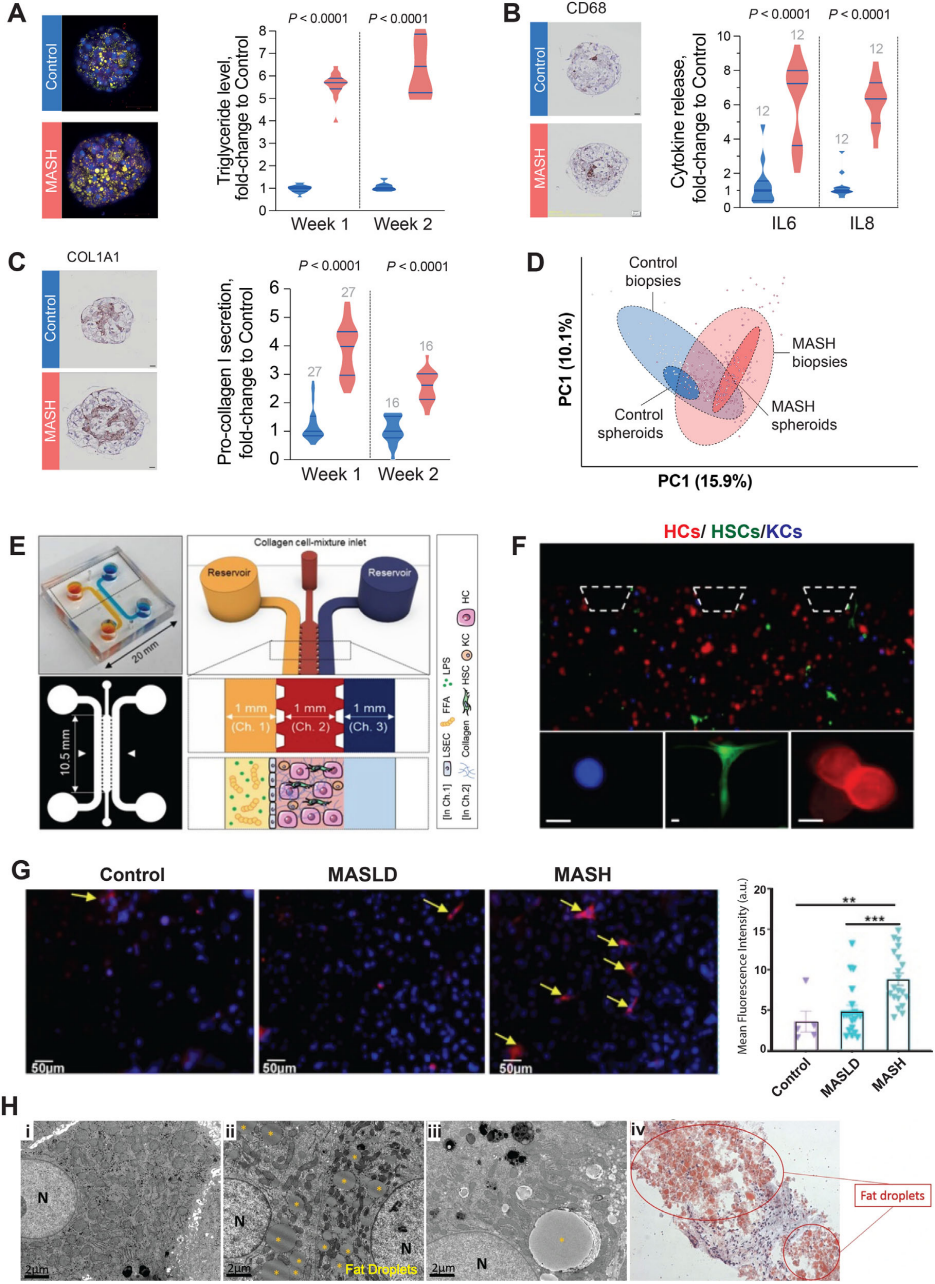
Representative human in vitro models of MASH. A–D) Spheroid cultures from MASH patient‐derived liver cells recapitulate key disease hallmarks. Illustrations reproduced with permission.^[^ ^64^ ^]^ Copyright 2025, Wiley. A) Intracellular lipid accumulation (Nile red staining) and triglycerides quantification shows a significant increase in MASH spheroids compared to controls. B) Pro‐inflammatory cytokines secretion is significantly elevated in MASH. C) MASH spheroids exhibit significantly increased collagen deposition and pro‐collagen secretion in MASH versus control. D) Principal component analysis (PCA) of transcriptomic signatures shows that the MASH defining alterations in expression patterns are reproduced ex vivo. Note that the heterogeneity that exists between human patient and control biopsies is maintained in liver spheroids. E–G) MASH‐on‐a‐chip model. The respective figure panels are reproduced with permission.^[^ ^123^ ^]^ Copyright 2021, Wiley. E) Model schematic depicting a central hydrogel channel embedded with hepatocytes (HCs), Kupffer cells (KCs), and hepatic stellate cells (HSCs), bordered by inlet and outlet channels lined with liver sinusoidal endothelial cells (LSECs). F) Microscopic image of the central collagen compartment containing both parenchymal and non‐parenchymal cell types; scale bars are 25 µm. G) Representative fluorescence images and mean fluorescence intensity quantification of COL1A1 staining under control, MASLD and MASH conditions after 10 days of disease induction. H) Fat accumulation in human precision‐cut liver slices treated with a combination of free fatty acids (0.1 mm oleic and linoleic acids) for 24 h. Lipid accumulation within hepatocytes visualized using electron microscopy in control i) and fatty acid treated cuts ii, iii) showing hepatocyte steatosis. iv) Light microscopy following showing microvesicular and macrovesicular steatosis by Oil Red O staining. Reproduced with permission.^[^ ^130^ ^]^ Copyright 2019, Springer Nature.

Recently, a 3D spheroid model was presented in which cells from patients with histologically confirmed MASH were cultured.^[^ ^64^ ^]^ Comprehensive transcriptomic, proteomic, phosphoproteomic and lipidomic profiling complemented with extensive functional and histological assessments confirmed that these spheroids recapitulated key hallmarks of the disease, including steatosis, hepatocyte ballooning, elevated pro‐inflammatory cytokines, and robust ECM remodeling (**Figure** **2**A–C). Moreover, transcriptomic comparison to liver biopsies from 306 MASH patients demonstrated that the model carried over the inter‐individual heterogeneity that constitutes an intrinsic feature of the disease (Figure 2D), thereby allowing for investigations into different disease stages and genetic predispositions.

#### 3.2.2 Microphysiological Systems

Microphysiological systems (MPS) integrate microfluidic technologies, biological scaffolds, and organotypic tissue models comprised of primary mature cells or stem cells, often with various biological scaffolds, to recapitulate dynamic tissue microenvironments. MPS enable precise spatiotemporal control of shear stress and cellular exposures to nutrients and pharmacological agents. Leveraging these possibilities by introduction of dynamic perfusion regimens involving metabolic modulators, such as glucagon and insulin, allows to induce distinct metabolic regions in the MPS, recapitulating zonation‐like phenomena.^[^ ^113^ ^]^ Similarly, introduction of oxygen gradients showed that hypoxia enhanced lipid accumulation in hepatocytes under moderate free fatty acid exposure.^[^ ^114^ ^]^

A multitude of different liver‐on‐a‐chip (LoC) platforms have been developed to date. These are constructed through techniques such as soft lithography, micropatterning and 3D bioprinting, and often include multilevel fluidic channels capable of supporting co‐cultures of parenchymal and non‐parenchymal liver cells. Advanced platforms also incorporate oxygen sensors,^[^ ^115^ ^]^ endothelial barriers^[^ ^116^, ^117^ ^]^ and real‐time sampling of circulating media, allowing for dynamic monitoring of disease progression and therapeutic responses. In the context of MASLD, LoC systems commonly utilize the administration of lipotoxic media comprised of free fatty acids, as well as high glucose and insulin concentrations. Such models can be generated from cell lines,^[^ ^118^, ^119^ ^]^ differentiated stem cells^[^ ^120^ ^]^ or primary mature cells.^[^ ^121^, ^122^, ^123^ ^]^ Notably, complex MPS comprising different cell types are able to mimic aspects of disease progression, such as the increasing deposition of collagens upon prolonged nutritional stimulation (Figure 2E–G).

Unique to MPS is the possibility to connect different tissue models in an interconnected fluidic circuit. In the context of MASLD, mature human liver cells were cultured together with human adipocyte‐like cells in two separate compartments of a microfluidic chip. In one study, mature human hepatocytes and adipocytes differentiated from primary human preadipocytes were cultured as monolayers.^[^ ^124^ ^]^ Despite the rapid decline in phenotypes in monolayer culture, this model successfully recapitulated in vivo cytokine‐driven lipotoxicity evidenced by the observation that TNFα did not induce steatosis in hepatocyte monocultures but did so robustly in the presence of adipocytes. In another study, both HLCs and adipocyte‐like cells were differentiated from stem cells and supplemented with isogenic macrophages to model inflammatory interplay in metabolic dysfunction.^[^ ^125^ ^]^ It was demonstrated that inflammation rather than mere expansion of adipocytes was the primary driver of hepatic insulin resistance and lipid accumulation. Furthermore, the authors showed that the GLP1 agonist semaglutide reduced hepatic steatosis and insulin resistance through its action on adipocytes, underscoring the importance of ameliorating adipose tissue inflammation for the treatment of metabolic aberrations in other tissues and organ systems.

MPS drastically reduce throughput and increase experimental complexity and costs. To outweigh these disadvantages, it is important to make the most out of the strengths of the models. This requires that the modeled tissues are constructed from cells with physiologically relevant phenotypes using culture methods in which their functionality can be maintained for sufficient periods of time. Furthermore, integration of biosensors, high‐resolution imaging, and advanced analytical modalities can increase data yield by enabling longitudinal profiling. For instance, amperometric glucose and lactate biosensors embedded in liver‐on‐chip devices enable real‐time non‐invasive metabolic monitoring, providing continuous insight into hepatocyte metabolism.^[^ ^126^ ^]^ Similarly, albumin sensors have been incorporated to dynamically track hepatic functionality under hypoxic stress.^[^ ^127^ ^]^ Moreover, multisensor arrays combining oxygen and pH sensors with impedance spectroscopy and label‐free imaging can enable simultaneous monitoring of zonation, barrier function and bioenergetic states.^[^ ^128^, ^129^ ^]^ These integrated technologies thus provide continuous, quantitative and physiologically relevant readouts of tissue model dynamics, which is exclusively achievable in MPS. However, while many of the aforementioned systems show encouraging results, MPS remain at present mostly at the research stage and their application in projects with a translational scope requires further standardization, in‐depth characterization and benchmarking.

### 3.3 Precision‐Cut Liver Slices

Originally introduced in the 1980s as a physiologically relevant alternative to hepatocyte monolayer cultures, precision‐cut liver slices (PCLS) are by now also used modeling liver physiology, chronic liver diseases, and drug‐induced toxicity.^[^ ^130^, ^131^ ^]^ The advantages of PCLS lie in their ability to retain the full complement of hepatic cell types, which also includes infiltrating immune cells, within their native ECM and tissue microenvironment. This makes them well positioned to recapitulate the multicellular interactions in MASLD and MASH, for instance upon exposure to steatogenic culture media (Figure 2H). PCLS are prepared from partial hepatectomy material, or resections from fibrotic or peritumoral liver tissue in a process that involves rapid excision and cold perfusion followed by embedding in low‐melting‐point agarose and precision slicing—typically 150–250 µm thick—using a vibratome. Viability in these slice cultures can be preserved for up to 5–7 days.

Several recent studies have demonstrated the use of PCLS for investigating MASLD mechanisms and the evaluation of therapeutic opportunities. For instance, PCLS were used to evaluate the functional role of VAP‐1, a hepatic amine oxidase, in promoting steatosis and inflammation.^[^ ^132^ ^]^ Treatment of PCLS from healthy livers with recombinant VAP‐1 and its substrate methylamine led to increased lipid accumulation and reduced triglyceride secretion, suggesting that VAP‐1 promotes intracellular lipid retention. Inhibition of VAP‐1 with bromoethylamine attenuated both steatosis and inflammatory responses, indicating that targeting the oxidase activity of VAP‐1 may offer dual benefits for managing steatosis and inflammation.

For later disease stages, PCLS have contributed to the identification of specific T cell subtypes in human liver fibrosis. Using slices from patients with end‐stage fibrosis, mucosal‐associated invariant T (MAIT) cells were identified to be spatially clustered near activated HSCs.^[^ ^133^ ^]^ Notably, inhibition of MAIT activation with acetyl‐6‐formylpterin (Ac‐6‐FP) led to marked downregulation of inflammatory and fibrotic markers, such as IL1B, TGFB1 and CCL2, as well as to a reduction in αSMA^+^ cells. These results indicate that pharmacological blunting of MAIT cell activation may present a novel antifibrotic strategy in fibrotic MASH. In a more direct example of the application of PCLS to drug development, AZD3355, a GABA‐B receptor agonist originally developed for gastroesophageal reflux, was tested as a candidate antifibrotic therapy in MASH.^[^ ^134^ ^]^ Treatment of fibrotic PCLS from four patients with AZD3355 for 24h significantly reduced the expression of αSMA and TNFα, as well as COL1A1 secretion. The authors conclude that PCLS provide a useful puzzle piece for the rapid repurposing of drugs and drug candidates.

Despite these advantages, several limitations restrict broader application of PCLS. First, the need for freshly resected human liver tissue inherently limits sample accessibility. Particularly for early‐stage MASLD patients this means that samples can only be obtained incidentally if patients undergo hepatic resection for a different indication. Second, the overall stability of the sections declines rapidly ex vivo, limiting exposure times to few days. This limitation is particularly problematic for chronic diseases, such as MASH and fibrosis. Lastly, culture conditions, such as oxygenation, nutrient composition and perfusion rates are not yet standardized across laboratories, which can affect reproducibility and limit cross‐study comparisons.

### 3.4 Bioprinted Liver Constructs

Bioprinting has emerged as a transformative approach in liver tissue engineering, offering unprecedented spatial control and structural fidelity. This technology enables the precise deposition of the different hepatic cell types within a scaffold in an attempt to replicate the liver's intricate sinusoidal architecture. In the context of MASLD, there have been promising developments at the level of bioinks used to encapsulate cells during deposition and the printed designs. For bioinks, a set of bioinspired hydrogels (termed s‐Hep3Gel) for the fabrication of chemomechanically relevant models of hepatic steatosis using extrusion‐based 3D bioprinting were presented.^[^ ^135^ ^]^ These alginate‐based hydrogels were engineered to recapitulate the viscoelastic properties of steatotic liver tissue and incorporated porcine liver‐derived ECM and sodium oleate to provide biochemical cues and trigger intracellular lipid accumulation, respectively. The authors demonstrated that the bioinks could reliably form structurally stable constructs and induced concentration‐dependent lipid droplet formation in HepG2 cells. However, the inks have not yet found wider dissemination.

For bioprinted tissue constructs, MASH‐like phenotypes can be induced upon exposure to media containing free fatty acids, high carbohydrate levels and LPS. In models comprising primary hepatocytes, Kupffer cells, HSCs and LSECs from individuals without MASH, exposure to such a cocktail resulted in steatosis, inflammation and fibrosis, as well as in transcriptomic alterations that approximated changes observed in MASH patients.^[^ ^136^ ^]^ Notably, in constructs printed from MASH patient‐derived cells, the authors show that the MASH phenotype does not require the addition of pro‐inflammatory cues, such as LPS.^[^ ^137^ ^]^ These results provide further evidence for the importance of using patient‐derived cells for the investigation of disease mechanisms and therapeutic interventions.

Several challenges continue to limit the broader application of bioprinted liver constructs. First, bioprinting is associated with high costs, both in terms of infrastructure investments and high consumable costs for proprietary bioinks, which overall limits accessibility and widespread adoption of this technology. Second, scalability and reproducibility remain major hurdles ‐ while small constructs can be reliably produced, printing large or vascularized tissues with uniform cell distributions and long‐term viability is still difficult. Additionally, the lack of standardization with regards to bioinks and protocols complicates cross‐study comparisons and raises concerns about consistency in disease modeling. Finally, while protocols have been developed that ameliorate cell stress during printing, the maintenance of functional phenotypes of bioprinted liver constructs over prolonged culture periods remains unresolved. Due to these challenges, the application of 3D bioprinting to MASLD research remains in its infancy.

## 4 Use Cases of 3D Human Liver Models in Drug Discovery and Development

The increased physiological relevance of 3D human liver models facilitates their meaningful application for the testing of toxicity and efficacy. In vivo, MASLD constitutes a significant risk factor for drug‐induced liver injury (DILI), likely due to alterations in pharmacogene expression and/or ongoing hepatocyte injury due to chronic oxidative stress and a pro‐inflammatory milieu.^[^ ^138^, ^139^ ^]^ Since advanced MASLD and MASH models have the potential to capture disease‐relevant alterations in cellular, molecular and functional parameters, this opens the possibility to emulate impacts of MASLD on drug disposition and hepatotoxicity. However, while it is plausible that MASLD models could help to predict the increased sensitivity of MASLD patients to compounds of interest earlier in the drug development process, no conclusive data to this effect has been presented to date.

3D human liver models have been used in a number of screening campaigns. Screening of 60 compounds in a MASH model of human liver organoids from differentiated iPSCs, resulted in the identification of multiple antifibrotic compounds.^[^ ^140^ ^]^ As top hit, the authors identified the selective TGFβ receptor inhibitor SD208, which was shown to be effective not only when fibrosis was induced by TGFβ, but also by methotrexate and LPS. Imatinib, a pan‐tyrosine kinase inhibitor and clinically approved for use in various malignancies, was identified as a second confirmed hit. At the concentration used (1 µm and 100 nm), imatinib has about a dozen molecular targets and it remains unclear what the main cellular target for the observed anti‐fibrotic activity is. It would further strengthen the translational insights of these encouraging results if the molecular target responsible for this effect could be identified, e.g. by dissecting the molecular mechanisms with chemogenomic kinase sets or available chemical probes.^[^ ^141^ ^]^

Genetic screens can serve as a powerful orthogonal approach to pharmacological tests. In a CRISPR‐Cas9 screen of 35 human fetal hepatocyte organoids, FADS2 was identified as a key determinant of hepatic steatosis.^[^ ^36^ ^]^ Interestingly, comparison of PNPLA3 knock‐out organoids with isogenic knock‐in PNPLA3 p.I148M and wildtype organoids showed that both knockouts and knock‐ins exhibited increased steatosis in contrast to the murine knockout model that does not induce steatosis.^[^ ^142^ ^]^ These data reinforce the need for human cell models for studies of genetic predisposition in metabolic disorders. From a translational perspective, these organoid results do not match the observations from clinical trials, which showed reduced liver fat content upon treatment with GalNac‐conjugated antisense oligonucleotide drugs against *PNPLA3*, such as AZD2693.^[^ ^143^ ^]^

In an organoid model based on iPSC‐derived HLCs, the authors used the Tocrisscreen Mini library containing a diverse set of biologically active compounds using the size and accumulation of lipid droplets as parameters for steatosis.^[^ ^75^ ^]^ The library was screened at 5 µm, exploiting the strength of well‐characterized chemogenomic compound sets. Among the confirmed hits were Src, PI3K and AKT kinase inhibitors with supposedly overlapping activity. Indeed, when sourcing orthogonal chemotypes in a chemogenomic approach, all primary hits and new members of the set showed a markedly decreased droplet size and fat accumulation at specific low concentrations of compound. These results suggest that the Src and Akt axis might be mechanistically promising ‐albeit therapeutically likely unviable ‐ target for anti‐steatotic drug development.

We recently presented a 3D liver model based on cells from patients with histologically confirmed MASH and evaluated 16 drug candidate that were under clinical development at the time of the experiments. Among the six drugs that showed improvement in liver fibrosis were resmetirom, which was approved for MASH/fibrosis after a successful phase 3 randomized controlled trial^[^ ^23^ ^]^ and aramchol, which just showed reduction in liver stiffness and improved fibrosis in an interim analysis of phase 3 results.^[^ ^144^ ^]^ In contrast, cenicriviroc, selonsertib and elafibranor, which all ultimately failed in clinical development did not exhibit beneficial effects on hepatic fibrosis. Investigation of the latter at the systems level using integrative omics analyses showed that elafibranor reduced steatosis and inflammation but not fibrosis, aligning with clinical findings.

Oxidative stress drives mitochondrial dysfunction and DNA damage and in MASH is linked to polyploidization.^[^ ^145^ ^]^ Evaluation of fibrosis levels in MASH spheroids upon treatment with a compound set designed to modulate DNA damage repair and bioenergetics, revealed three organocatalytic switches (ORCAs) of OGG1 among the hit molecules.^[^ ^146^, ^147^ ^]^ Investigating one of the hits, TH10785, in more detail showed a reduction in oxidative DNA lesions in agreement with the proposed mechanism of action. Targeted proteomics furthermore confirmed reduced levels of the fibrotic markers TGFB1, COL1A1 and MMP7 as well as of pro‐inflammatory cytokines. These results were corroborated using optimized derivatives of TH10785, which further improved on the antifibrotic effect of ORCAs.^[^ ^148^ ^]^ Combined, these results suggest that phenotypic multimodal drug testing in patient‐derived organotypic MASH models can accurately predict anti‐fibrotic effects of drugs and drug candidates and provide translational insights in the mode of action of the tested intervention.

## 5 Conclusion

Given the multifactorial etiology and pathogenesis of MASLD, accurate emulation of all disease‐relevant mechanisms in vitro remains challenging. Careful characterization and benchmarking of models at the omics level, ideally in multi‐center cross sectoral trials, are therefore essential to bridge the translational gap that currently impedes the progression from preclinical discovery to clinical intervention. Importantly, the use of patient‐derived cells not only improves disease relevance but also supports the identification of biomarkers and the development of precision therapies that account for inter‐individual variability in treatment response. These prospects are enabled by the integration of these models with next‐generation technologies, such as spatial transcriptomics, single‐cell multi‐omics, biosensor‐enabled feedback systems, and machine learning‐based image analysis that significantly enhance model resolution and amplify the mechanistic insights.

Importantly, both the EMA and FDA signal increasing openness to data generated by organotypic and microphysiological models for investigational new drug filings and marketing authorization applications. Particularly, for primary pharmacology studies related to the target or mechanism of action of a candidate molecule, requirements are not very stringent and such data is already well admissible in regulatory application packages. Furthermore, in 2022 the FDA Modernization Act 2.0 paved the way to include preclinical in vitro methods even as part of a safety package.^[^ ^149^ ^]^ More recently, in 2024, the FDA Modernization Act 3.0 instructed the FDA to develop clear guidance on how to qualify the suitability of organotypic and microphysiological models for predictive preclinical investigations.^[^ ^150^ ^]^ These developments emphasize the importance to accurately characterize new model systems to demonstrate they are translationally relevant and fit‐for‐purpose; however, in the context of the liver, characterization of most current model systems is sparse and only few systems have been compared at the molecular, cellular and functional level to in vivo tissue and patient‐derived phenotypes. This highlights the responsibility of all stakeholders, including model developers, publishers, drug developers and regulators to prioritize careful characterization and benchmarking studies to set the stage for model systems that contribute to increased clinical success rates in a real‐world setting.

Importantly, despite their increasingly relevant phenotypes, even the most advanced liver models must remain reductionistic in nature, thus uncoupling hepatic manifestations of MetS from the systemic effects. This provides both opportunities and limitations. It opens new possibilities for mechanistic liver research and drug development but also critically requires that the resulting data is complemented and contextualized with data on systemic effects. Thus, despite substantial political impetus, it is at present difficult to envisage that in vivo testing in MASLD research can be fully replaced by in vitro methods. The increasing possibilities to emulate the metabolic effects of extrahepatic tissues on liver phenotypes might however pave the way for a further reduction of animal use. This shift in perspective calls for multi‐organ modeling strategies that incorporate adipose tissue, intestinal models or circulating immune cells into MPS to better capture disease pathophysiology and mimic metabolic interactions and their pharmacological modulation across organ systems. Close interdisciplinary collaboration between bioengineers, data scientists, pharmaceutical developers and clinicians will be pivotal in realizing this vision. By advancing the design, standardization, and integration of complex organotypic and microphysiological human models, the field is poised to accelerate the discovery of effective therapies and improve therapeutic opportunities for the millions of patients living with MASLD worldwide.

## Conflict of Interest

V.M.L. is CEO and shareholder of HepaPredict AB, as well as co‐founder and shareholder of Shanghai Hepo Biotechnology Ltd. The other authors declare no competing interests.
